# Predicting patient enrollment in a telephone-based principal care management service using topic modeling

**DOI:** 10.1371/journal.pdig.0000992

**Published:** 2025-09-18

**Authors:** Annisa Marlin Masbar Rus, Julie S. Ivy, Min Chi, Mitchell Plyler, Elaine Wells-Gray, Maria E. Mayorga

**Affiliations:** 1 Department of Industrial and Systems Engineering, North Carolina State University, Raleigh, North Carolina, United States of America; 2 Department of Computer Science, North Carolina State University, Raleigh, North Carolina, United States of America; 3 Lumata Health, Oklahoma City, Oklahoma, United States of America; London School of Hygiene and Tropical Medicine, UNITED KINGDOM OF GREAT BRITAIN AND NORTHERN IRELAND.

## Abstract

Diabetic Retinopathy (DR) is a complication related to diabetes that can lead to vision impairment. To assist DR patients, a care management company provides a telephone-based principal care management (PCM) service, which includes care coaching and other services to reduce barriers to care for patients with DR. Despite its benefits, enrollment in the program is suboptimal. This study developed predictive models using call transcripts to investigate factors associated with patient enrollment in the PCM service. We analyzed transcripts of calls made during the enrollment process (prior to enrollment) and feature-engineered the call metadata (i.e., transcript length, number of calls, time between calls, customer and agent sentiment). In addition, we extracted topics discussed in the transcripts using Structural Topic Modeling (STM) and converted them into vector representations. Utilizing call metadata alongside topics, we developed three classification models (call metadata, topic-based, and topic+metadata) to predict patient enrollment, with the latter demonstrating superior performance. The topic+metadata classification model outperformed the other two models in distinguishing between patient enrollment and non-enrollment, with AUC values ranging from 0.81 to 0.99 across models using 3 to 15-topics. The findings suggest that proactively offering to schedule an appointment after the program benefits explanation leads to a higher odds of enrollment. When the scheduling portion of the conversation is not considered, agents should cover all parts of the script over multiple calls. Additionally, agents who explain the program and maintain longer intervals between calls have higher odds of patient enrollment, suggesting that there is value in allowing patients adequate time to reflect between calls. These findings offer valuable insights for agents to evaluate their strategies in patient enrollment. As the first point of contact, enrollment agents play a crucial role in determining whether patients can benefit from care coordination and management programs.

## Author summary

Diabetic retinopathy (DR) is a diabetes-related complication that can lead to blindness. Annual eye exams for diabetic patients are crucial for early detection and risk reduction. A telephone-based principal care management (PCM) service offers coaching and assistance to help patients access care, but enrollment in the program is low. We identify the factors that influence enrollment by analyzing transcripts of the conversations between the patient and the PCM agent. These factors include the transcript length, number of calls, time between calls, customer and agent sentiment, as well as the topics discussed. We automatically identified the topics discussed by analyzing groups of unique words that are most likely to occur together in the transcripts of patient-agent conversations and interpreting them into various topic names, such as “program explanation.” After that, we analyzed these factors and found that simply explaining the service to patients is not sufficient. Calls in which agents explain the service and proactively offer to schedule a coaching call are more likely to lead to enrollment. Furthermore, more time between follow-up calls also leads to better enrollment outcomes. Our study provides valuable insights for agents that provide care management services to evaluate their strategies for patient enrollment.

## Introduction

Diabetic Retinopathy (DR) is a diabetes complication that can lead to Vision Threatening Diabetic Retinopathy (VTDR) and even blindness [1]. Detecting VTDR can be challenging due to its slow progression; thus, it is recommended that DR patients visit an eye doctor annually to ensure early detection [1]. Unfortunately, in 2020, only 58.3% of adults with diabetes received an eye exam, falling short of the 70.3% target set by Healthy People 2030 [2]. This concern has been labeled as “worsening" by the U.S. Department of Health and Human Services [2].

One favorable approach to help patients manage their eye care is through telephone-based health coaching (TBHC) and counseling. Previous research on patients with diabetes indicates that TBHC is cost-effective and can assist patients with physical activity and dietary management, but the implementation of these programs varies, such as having different call frequencies or coaching content [3–7]. A US study involving 174,120 patients with various chronic conditions, including diabetes, also showed that generic TBHC with shared decision making, self-care and behavioral change instruction from the health coaches can reduce hospitalizations, surgeries and healthcare costs [8]. In a separate investigation, Wungrath and Autorn [9] observed that telephone-based counseling effectively enhanced medication adherence in patients with type II diabetes.

One such TBHC program that focuses on providing care coaching for VTDR is provided by a care management company. We refer to this as principal care management (PCM) service throughout the rest of the manuscript. This service, led by a certified ophthalmic assistant (COA), supports patients in managing their care through phone calls in between doctor visits. The COA could help patients with financial assistance programs, prescriptions and refills, insurance issues, transportation to visits, understanding their disease and care plan, and coordinating care with other providers. However, despite the service benefits, around half of the doctor-recommended patients did not enroll in the PCM service. The objective of this study is to explore factors associated with patients’ decisions to either participate in or abstain from enrolling in the PCM service.

Other researchers have explored factors associated with enrollment. Haynes et al. [10] qualitatively explored a coaching program promoting physical activity for older people. They explored the factors that contribute to low participation by performing a secondary thematic analysis of semi-structured interview data. However, this approach may overlook certain interview topics and relies on participants’ recall memory. Therefore, to mitigate these concerns, this study adopted a distinct approach by analyzing all of the conversational data (i.e., call transcripts) directly to extract relevant themes related to patients’ enrollment in the PCM service.

Given the substantial size of the data, employing natural language processing (NLP) for automated analysis of text could be beneficial in exploring the call transcripts. The PCM company automatically transcribed the call interactions between patients and COA agents for program enrollment purposes. Reading these transcripts would be time-consuming. Thus, we employ NLP on these call transcripts to explore factors associated with not enrolling in the PCM service. In this study, we propose to achieve this objective by transforming the call transcripts into vector representations which can serve as features for predicting program enrollment.

Call transcripts present challenges compared to other text documents due to their non-continuous nature, the inclusion of irrelevant “small talk," and grammatical errors [11]. However, these concerns are alleviated when employing topic modeling, such as Latent Dirichlet Allocation (LDA), which operates at the word level. Using topic modeling, transcripts can be transformed into vector representations and used as features for prediction tasks. A few studies explored the use of call transcripts for prediction, but they lack interpretability due to the black-box approaches used (e.g., convolutional neural networks), making it difficult to identify the content or topics associated with the call outcome [12,13]. In contrast to black-box approaches, the topic modeling approach allows us to pinpoint the specific extracted topics that influence patient enrollment decisions.

Numerous healthcare studies have leveraged topic modeling to vectorize documents, such as clinical notes, and have paired the results with electronic health record (EHR) data, including demographic information, ICD-9/10 diagnosis codes, and laboratory tests, to facilitate prediction. These studies have demonstrated promising performance in predicting mortality in different timelines, diagnosing HIV, and predicting future falls in older people [14–16]. Rijcken et al. [17] investigated various topic modeling approaches, such as LDA, specifically for prediction purposes. Structural Topic Modeling (STM) is a newer technique introduced by Roberts, Stewart, and Airoldi [18] that has received less attention in research compared to LDA. LDA does not consider the correlation between topics, whereas STM takes into account both the correlation between topics and document metadata, such as authorship, when generating topics. This study intends to investigate the atypical text format of call transcripts and explore the previously underexamined topic modeling method, STM, to produce document features for prediction.

In brief, the study objective is to explore factors associated with patient enrollment in a telephone-based counseling program, the PCM service, from call transcripts collected during the enrollment process. To achieve this objective, we transformed enrollment call transcripts into vector representations by using STM to generate extracted topics that serve as document features. Subsequently, we predict enrollment by utilizing both these topics and call metadata and evaluated the predictive performance of three classification models.

## Methods

This section will provide detailed information regarding the Principal Care Management (PCM) service considered in this study, transcript data and the inclusion criteria, the chosen call metadata as predictive factors, an overview of topic modeling, and the configuration of the prediction model settings. Table 1 presents a visual representation of the overall research workflow. This study has been approved by the North Carolina State University institutional review board (IRB) under protocol # 12184.

**Table 1:** Overall flow of the methods used.

Workflow	Details
**Step 1. Data preparation**	The following are the data inclusion criteria: • Filter patients based on their presence in coaching calls after enrolling in the program. • Filter calls based on the transcript availability and call length.
**Step 2. Call metadata preparation**	Call metadata that are used as predictors include: • **Total transcript length** in combined calls per patients • **Number of calls** received by patients in enrollment process. • **Interval** of time between the last call and the call before in days. • **Maximum of agent sentiment** • **Minimum of patient sentiment**
**Step 3. Text preprocessing**	The objective of this phase is to identify significant words that can be used for topic modeling. The text preprocessing includes: • Converting text to lowercase • Removing stopwords, such as I, am, and you. • Lemmatizing words or converting the words into root words. • Tokenizing words.
**Step 4. Topic Modeling**	In this phase, we applied Structural Topic Modeling (STM) to incorporate enrollment status in extracting topics from the call transcripts. • Metadata that is used is the enrollment status (1 = enroll, 0 = not enroll) • Number of topics analyzed is from 3 to 15 topics.
**Step 5. Classification models**	Model details : • Logistic regression with Lasso is our classifier. • All independent variables are normalized. • Train and test data are split into 0.9 and 0.1, respectively. Models: • **Metadata model**: using call metadata as predictors. • **Topic-based model**: using topic proportions generated from STM as predictors. • **Topic + metadata model**: using topic proportions and call metadata as predictors.
**Step 6. Performance Evaluation**	In this phase, we evaluate the coefficients and the models. • For the coefficients, we do not evaluate individual coefficients using p-values because Logistic Regression with Lasso artificially shrinks them, rendering them biased. • For the models, we will evaluate them using the Friedman’s test to identify which specific pairs of models exhibit statistically significant differences in performance.

### Principal Care Management (PCM) Service

The PCM service provides telephone-based care coaching with certified ophthalmic assistants (COA) for patients with chronic eye conditions, aiming to bridge the gap between doctor’s appointments and eye care management. In contrast to telephone-based health coaching and counseling programs for patients with diabetes, which provide general advice on physical activity and diet, this program specifically focuses on the patient’s eye care management, including diabetic retinopathy, and assists patients with logistical issues (e.g., booking appointments, prescription management, and arranging transportation to appointments).

The PCM service enrollment process begins with a doctor’s appointment, during which the physician may recommend the program if deemed beneficial. Subsequently, a PCM service agent contacts the patients via phone to provide program details and extend the offer. Patients who consent to participate will receive future calls from the COA based on patients’ availability. We refer to these interactions before the counseling begins as “enrollment calls”, which are the focus of our study.

### Data

#### Call inclusion criteria.

**Fig 1:**
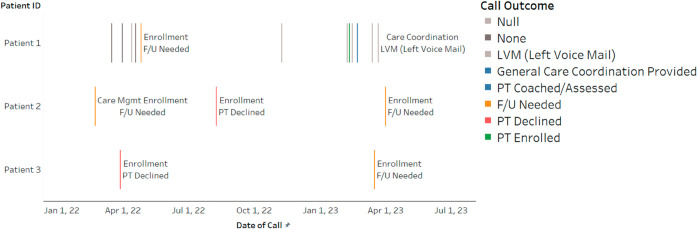
Call history example. Examples of three patients’ call history over time. The y-axis shows the patient ID, and the x-axis shows the date. Each patient has different colored bars that indicate different call outcomes. Each bar is labeled with the call type indicated on the first line of the label and the call outcome on the second line. However, not all calls in this figure are labeled due to the proximity of the lines.

For this study, we relied on transcripts of enrollment calls as our primary text data for topic modeling. These calls were recorded, transcribed automatically using Amazon Web Services (AWS), and reviewed by the care management agency to remove any personally identifiable information (PII). Furthermore, to ensure confidentiality, the dates of the calls were shifted for each patient, while maintaining the sequence and time between calls. It is worth noting that patients may have multiple calls before making a decision to enroll or decline participation in the PCM service, as depicted in Fig 1. The recorded enrollment calls span from June 7th, 2021 to April 13th, 2023, and comprise 30,662 calls, all in English.

We identify each call with a call ID, and its outcome is manually perceived and recorded by the program agent after the conversation, referred to as the “call outcome." For this study, we will only consider four distinct call outcomes related to enrollment calls. Other outcomes related to voicemail messages, transfers, calls disconnected, busy, leaving a message with someone, or invalid numbers will be excluded. However, we include the frequency of these calls in the total count of the number of calls. The four relevant outcomes are “Patient Enrolled" (PT Enrolled), “Patient Declined" (PT Declined), “Patient Unenrolled" (PT Unenrolled), and “Follow-Up Needed" (F/U Needed). For analytical simplicity, we have categorized these outcomes into two overarching groups: “enroll" (comprising PT Enrolled) and “not enroll" (comprising PT Declined, PT Unenrolled, and F/U Needed). Here, F/U Needed means the patient has not yet enrolled, the final outcome of the patient is determined by the last recorded call, as described below. Our dataset consists of a total of 9,382 call IDs meeting these specified criteria.

In this study, we further filter the text dataset (i.e., 9,382 call IDs) through a four-stage process.

1. **First stage: Participant selection**
  In the first stage, we applied a patient-level filter. Specifically, for patients who enrolled in the program, we focus on individuals who actively participated in the coaching program following enrollment, meaning that their enrollment was followed by a coaching call. This approach ensured that the included patients were deliberately interested in the program instead of merely expressing initial interest and subsequently disengaging from the agent. For patients who did not enroll, all calls were included.
2. **Second stage: Unavailable transcript exclusion**
  The second stage involved filtering based on transcript availability, as not all recorded calls were accompanied by transcripts. We only retained calls with available transcripts for analysis.
3. **Third stage: Short transcript length exclusion**
  In the third stage, we further refined the dataset by filtering calls based on transcript length. Because the distribution is skewed, we applied the log transformation to the transcript length and we excluded transcripts with log values in the lowest 10th percentile of the distribution. Consequently, we exclude calls with excessively short transcripts, which typically lack meaningful content for our analytical purposes, as well as calls that are unsuccessfully logged, transcribed, and answered. For instance, a call transcript containing minimal content, such as only containing the words “Okay, yeah, yeah.." was considered unhelpful for our analysis and removed. Such calls comprised 0.5% of the text dataset consisting of calls ranging from 1 to 31 words.
4. **Fourth stage: New patient exclusion**
  In stage four, we excluded newly called patients (last call outcome: “Follow-Up Needed”) contacted within the final two weeks of the study period. This ensured sufficient time for enrollment consideration after the initial call. A two-week window was chosen to exceed the company’s standard minimum call interval (one week), allowing for increased enrollment opportunities. We found 7 patients who met this criterion and excluded them from the analysis.

Ultimately, this four-stage filtering process resulted in a dataset of 6,741 call IDs, representing 72% of the initial call volume of 9,382 call IDs.

#### Call transcript.

The agent has a predefined script comprised of three sections, including an introduction, a program description, and a program enrollment offer. This document is available in the Supplement S1 File. In the introduction, the patient is informed that they received the call based on their provider’s recommendation. Typically, the agent will add some small talk in this section. Then, the conversation follows with an explanation about the PCM service that is delivered through phone calls and its overall benefit. In the event that the patient inquires about insurance coverage, relevant information regarding the patient’s coverage will be conveyed. Lastly, the agent will offer enrollment to the program. Should the patient express consent, prompt scheduling for the coaching process will be organized, while in the event of dissent, the agent will kindly request the patient’s motivation for their decision.

Note that each patient could receive multiple calls before they make an enrollment decision. To incorporate all calls, we sequentially organize the call history of individual patients according to call dates and consolidate all call transcripts into a unified transcript document for each patient. Additionally, we mark the last call outcome in the call history as the patient’s overall outcome, categorized as either “enroll" or “not enroll," following the categories described in the previous section.

#### Call metadata.

Apart from the transcript, we also include call metadata (information about the call) as features for our enrollment prediction task. By including the metadata, we could identify whether factors beyond the call’s textual content alone hold sufficient predictive power or whether the content also plays a significant role in shaping predictions. In this study, we utilize the following five types of metadata available to us:

1. **Total transcript length**. This is the cumulative word count from all transcripts in the patient’s call history. Henceforth in the document, when we refer to the patient’s transcript, we are referring to the combined transcript of all calls in the patient’s history. Total transcript length ranged from 32 to 15,000 words per patient.
2. **Number of calls**. The number of calls denotes the total number of calls received by patients, including voicemails. This count spans from 0 to 14 calls per patient. Note that here we include calls that went unanswered or were filtered due to transcript length in the total count.
3. **Interval**. Interval refers to the gap in days between the most recent call received by the patient and the penultimate call within the enrollment period. The interval values range from 0 to 500 days. A value of 0 means that the patient either received two calls on the same day or only received one call.
4. **Maximum of agent sentiment**. In this study, we use the overall sentiment value, which is automatically provided by Amazon Web Services (AWS). AWS uses machine learning (ML) methodologies, such as neural networks, to perform sentiment analysis with automated text extraction [19,20]. The sentiment score of individual responses per speaker are predicted and summarized as overall sentiment. For example, AWS labeled the customer’s response of “Alright. Yes. Yes, that’ll be fine. Thank you.." as positive with sentiment score 1.7. The overall sentiment is derived by averaging the sentiment scores within a given conversation. These overall sentiment scores range from -5 to 5.
  Our analysis focuses on the highest sentiment score expressed by the agent throughout calls with a particular patient. This approach allows us to assess the level of agent sentiment positivity associated with patient enrollment.
5. **Minimum of patient sentiment**. This is the minimum overall sentiment conveyed by the patient across calls, which also ranges from -5 to 5. We only consider the minimum of the patient’s overall sentiment throughout the calls received by the patient to assess the level of negativity associated with patient enrollment.

After conducting a thorough analysis of other metadata, such the percentage of follow-up and answered calls, we discovered that these other factors did not have as significant a predictive impact as the elements chosen. For results showing exploration of the use of other metadata, please refer to the Supplement S5 Appendix. Thus, only the five factors described above will be used as call metadata in the prediction models.

### Topic modeling

#### Text preprocessing.

As previously stated, our approach to processing call transcripts involves word-level analysis to overcome the challenges of handling non-continuous text. To prepare each patient’s transcript for analysis, we follow a standard procedure that includes eliminating URLs and punctuation, converting the text to lowercase, removing stop words, lemmatizing words, and tokenizing the text into individual words. We also remove redaction tags and rectify miss-transcribed words, such as changing “ice cream" to “eye screen." This method guarantees that we only consider relevant words for our bag-of-words approach to topic modeling.

#### Structural Topic Modeling (STM).

In this study, we used structural topic modeling (STM) as our topic modeling method [18]. STM is a derivative method from latent dirichlet allocation (LDA) by Blei, Ng, and Jordan[21], which is a well-known natural language processing (NLP) approach used in healthcare. As an unsupervised model, LDA extracts topics from a collection of documents or corpus. Each document is assumed to have a distribution of topics, and each topic is a distribution of words. For example, in a health article, the discussion of “disease symptoms" might be extensive, while “possible treatments" and “prevention of the disease" might be covered to a lesser extent, with the combined proportions summing up to one.

However, LDA does not consider topic correlations, which is concerning because the topic of “disease” is most likely related to “treatment”, for example. Thus, Blei and Lafferty[22] introduced correlated topic modeling (CTM) to address the issue. Then, STM extended CTM by incorporating metadata such as author’s identification [18]. In this study, we used patient enrollment status (i.e., enroll or not enroll). We ran the STM to generate topics from the transcripts that have gone through text preprocessing.

Determining the optimal number of topics to be modeled is a challenging and unresolved issue in topic modeling analysis. Roberts, Stewart, and Airoldi [18] consider the number of topics based on the combination of different measures, such as semantic coherence and word exclusivity within the topic group. However, an optimal number of topics does not necessarily translate to good predictors. Thus, we generated a series of models using 3 to 15 topics for comparison. Each patient transcript has non-zero proportion of topics for each topic model. For instance, for a 3-topic model, a patient’s transcript will have some proportion of topic 1, topic 2, and topic 3. Whereas in a 5-topic model, a patient transcript will have some proportion of topics 1 through topic 5, which sum up to one. We used these proportions as a vector representation for each patient transcript. These vectors are referred to as topic proportions and serve as features in the prediction models to predict enrollment.

We employed the FREX (Frequency and Exclusivity) metric to measure the exclusivity of words within topics [23]. FREX takes into account both the frequency of a word within a specific topic and its expected frequency across all other topics, thereby determining its level of exclusivity. It is calculated as the weighted harmonic mean of a word’s rank concerning exclusivity and frequency. As a result, we will have a list of words that are considered to be both frequent and exclusive to certain topic, referred to as FREX words. In the next section, we will explain the topic interpretation process based on their respective topic proportions and these FREX words.

#### Topic interpretation.

Generally, topic models, including STM, do not directly draw conclusions or present specific meanings of topics. Rather, the resulting topics represent groups of words frequently appearing together in documents. These word clusters enable humans to interpret them as topics based on their shared thematic associations. The ultimate goal of this interpretative process is to assign a representative label to each word cluster generated by topic modeling. This is accomplished by examining the FREX words within each topic and the text examples that best illustrate the respective theme (i.e., documents with a high topic proportion of a particular topic).

### Prediction models

We developed three models to predict enrollment outcomes in the PCM service (i.e., enrollment or not in the program). It is important to note that these models were not designed to predict the outcome of the next call but rather eventual enrollment based on the full call history so far. The first model relied solely on call metadata (metadata model), while the second model used topics derived from STM (topic-based model). The third model combined call metadata with extracted topics (topic + metadata model). We standardized the metadata and topic proportion values so that the mean is zero and the standard deviation is 1, following the assumption made in Tibshirani [24]. The standardization of variables was implemented to render them comparable by mitigating differences in scale and units.

We constructed each model using penalized logistic regression with the least absolute shrinkage and selection operator (Lasso), which allowed for variable selection during model fitting [24]. The fitting is conducted by shrinking some coefficients closer to zero or even to zero to filter out the variables that are considered unimportant. The method forces the coefficients to shrink towards zero by adding an L1 penalty to the loss function that encourages the coefficients to become smaller. Hence, the method forces the original or natural and unbiased coefficients to be smaller than the coefficients would be naturally. Since the coefficients are artificially manipulated and no longer represent their natural, unbiased values, we cannot accurately calculate p-values. Additionally, we cannot determine reliable confidence intervals (CIs) for these coefficients because the CIs would not be centered around the true parameter. While Lasso does not readily provide interpretable statistical significance measures like p-values, it prioritizes model accuracy and variable selection, leading to reduction in variance and improved out-of-sample prediction [24,25].

We evaluated individual predictors and interactions to understand their influence. Finally, we internally validated the models with 10-fold cross-validation and evaluated their performance based on the area under curve (AUC) of the the receiver operating characteristic (ROC) on the test data. To calculate confidence intervals (CIs) for the AUC we employ bootstrapping. Specifically, we create 2000 bootstrap samples by randomly selecting data points from our original dataset with replacement. Note that while we could also employ bootstrapping to calculate the CIs for the coefficients as suggested by Tibshirani [24], the bootstrap method leads the model to be inconsistent and unstable in selecting the variables [26]. Thus, we would not be able to test the significance of certain variables since the selected variables could be different in different bootstrap samples. Research regarding this topic is still ongoing. Thus, we provide the CIs of AUC only to give an estimate of the robustness of model performance.

All analyses were conducted using the R statistical software environment, with text processing performed using the “quanteda" package by Benoit et al. [27] and STM implementation carried out using the “stm" package by Roberts et al. [28]. Lasso and cross-validation were performed using the “caret" package by Friedman et al. [29] with “glmnet" method with α=1 (which indicates we use lasso regression).

### Evaluation criteria

In this study, we aim to evaluate and compare the performance of several prediction models on a single dataset. Our primary goal is to determine whether there are statistically significant differences in their performance metrics. We employ the non-parametric Friedman’s test due to potential violations of normality and independence assumptions to compare the models, as noted by Pizarro, Guerrero, and Galindo [30]. Unlike the Critical Difference Diagram (CDD) by Desmar [31], which is designed for comparisons across multiple datasets, the Friedman test is used because it focuses on identifying overall differences in performance rankings within our single dataset. If the Friedman test reveals a statistically significant difference in performance across all models (indicating at least one pair of models differs), we will proceed with a post-hoc test for pairwise comparisons. The Wilcoxon signed-rank test with Bonferroni correction is a suitable choice for this purpose. This test allows us to identify which specific pairs of models exhibit statistically significant differences in performance while accounting for the multiple comparisons problem arising from testing all possible pairs.

## Results

We obtained data on 6,748 calls from 5,698 patients, following the specified inclusion criteria and processing steps. The call history was arranged chronologically, and the outcome of the last call for each patient was recorded as either “enroll" or “not enroll". This resulted in a balanced dataset, with 49.77% of patients classified as “enroll" and 50.23% as “not enroll".

A summary of the call metadata values is provided in Table 2. The median aggregate transcript length for each patient is 815.5 words, with an interquartile range (IQR) of [489 - 791] (standard deviation (SD) = 861.32 words). Each patient received a median of 2.0 [IQR: 1 - 3] calls, with a standard deviation of 1.77. The median interval between the last call and the call before it is 0 days IQR [0 - 9.73] (SD: 62.60 days). Regarding sentiment, the median score of the minimum patient sentiment across the call history for each patient is 0.5 IQR [0 -1.1] (SD: 0.97) and the median of the maximum agent sentiment is 1.8 IQR [1.1 - 2.5] (SD: 1.00). Finally, all values were normalized using standard normalization to enable comparison between factors.

**Table 2:** The summary of the call metadata variables.

Call metadata (unit)	Median [IQR]			SD
	Enroll	Not Enroll	All	
Total transcript length (words)	1,054 [1,518 - 747]	569 [936 - 328]	815.5 [489 - 791]	861.32
Number of calls (calls)	2 [1 - 3]	2 [1 - 4]	2.0 [1 - 3]	1.77
Interval (days)	0 [0 - 7]	2 [0 - 11]	0 [0 - 9.73]	62.60
Minimum of patient sentiment	0.5 [0 - 1.1]	0.5 [0 - 1.2]	0.5 [0 - 1.1]	0.97
Maximum of agent sentiment	1.8 [1.1 - 2.4]	1.9 [1.1 -2.6]	1.8 [1.1 - 2.5]	1.00

### Extracted topics

Through the analysis of the call history of 5,691 patients, we generated topic models consisting of 3 to 15 topics with a unique mix of topic proportions for each document. These proportions were then utilized as features for classification and standardized to ensure comparability, so that the mean is 0 and the standard deviation is 1 [24]. Additional details on the topic proportion for each model can be found in the Supplement S1 Appendix.

### Predictive performance

**Fig 2:**
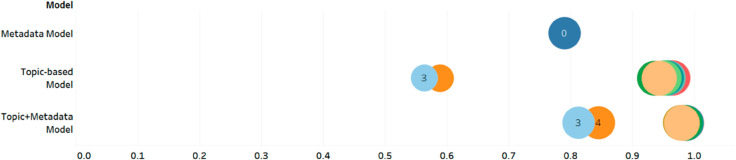
AUC performance. The AUC performance of metadata, topic-based, and topic + metadata models for 3 to 15 topic models. The x-axis is the AUC range from 0.5 to 1. Each circle represents each model’s performance and the number in the circle indicates the number of topics of each topic model. The number of topics is omitted from the topic models with 5 or more because the circles overlap.

Fig 2 displays the AUC performance of three model types in predicting enrollment using logistic regression with Lasso on the test set. The metadata model has only one model; the topic-based models consist of 12 models (ranging from 3 to 15 topics), and the metadata+topic models also have 12 models. The number in the circle indicates the number of topics used in the topic model.

The metadata model, which solely uses metadata as predictors, shows an AUC score of 0.79. The circle is labeled “0" because the metadata model does not use any topics. The topic-based models, which include only topic proportions as predictors, exhibit AUC scores ranging from 0.56 to 0.98. Only the models with 3- and 4-topics obtained AUC scores of less than 0.70, whereas the other models (5-15 topics) had higher AUC scores ranging from 0.97 - 0.98.

**Fig 3:**
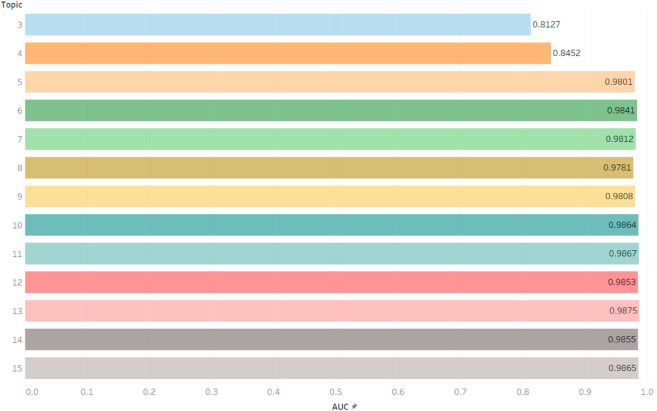
Detailed AUC performance of topic + metadata models. The AUC performance of topic + metadata model across 3 to 15 topics. The x-axis is the AUC range from 0.5 to 1. Each circle represents each model’s AUC performance.

In comparison, the AUC score in the topic+metadata models were generally higher than in the metadata and topic-based models. The AUC scores for the topic+metadata models using 3- and 4-topics increased to 0.81 and 0.84, respectively, while the AUC scores of the topic+metadata models with 5-15 topics ranged from 0.98 to 0.99. Fig 3 shows the detailed AUC performance for each topic+metadata model. Because the topic+metadata models have the same pattern across different number of topics and generally have higher AUC performance than the topic-based models, we will focus on the topic+metadata models in the next section. The CIs of the AUC of each model are found in Supplement (S2 Appendix). In general, we find that AUCs for each model are very robust. For example, for the 5 topic + metadata model, the 95% CI is 0.9704-0.9883.

### Evaluation

We employed a non-parametric Friedman’s test to assess potential differences in AUC between the predictive models on the test dataset. We treated topic-based models with different topic numbers as a single model category (i.e., topic-based model) and did the same for topic-metadata models. Thus, we treated different numbers of topics as different conditions. This resulted in a total of three model categories for comparison: metadata-only, topic-based (different number of topics considered as different conditions), and topic-metadata models (different number of topics considered as different conditions). The Friedman test revealed a statistically significant difference in ranking (${\tilde{\chi }}^{2}$(3) = 22.61, p ≤ 0.05) after Bonferroni correction for multiple comparisons (p adjusted = 0.0002 for all pairwise comparisons). This suggests that all 3 models (i.e., metadata-only, topic-based, and topic+metadata models) likely exhibit variations in their effectiveness on this task.

### Call metadata and topics associated with enrollment

In this study, we utilized the odds ratio (OR) to examine the relationship between predictors and outcome status. To account for varying variable scales, we normalized the value and interpreted the OR based on its magnitude rather than its actual value. A higher odds ratio indicates a stronger association between the predictor and outcome.

Our analysis of the metadata model revealed that metadata positively associated with patient enrollment in the program had an OR range of 1.01 to 3.42, while negatively associated metadata had an OR range of 0.65 to 0.99. We have presented the top five metadata positively and negatively associated with enrollment in Table 3 to highlight significant metadata and their interactions. Additional odds ratios for the metadata model can be found in Supplement S3 Appendix As per the table, the highest odds ratio belongs to total transcript length (OR 3.42), while the lowest belongs to the number of calls (OR 0.65).

**Table 3:** The top five variables exhibiting the highest coefficients and odds ratios, and the bottom five variables displaying the lowest coefficients and odds ratios within the metadata model.

Predictors	Coefficient	Odds Ratio (OR)
Top 5		
Total transcript length	1.229	3.418
Total transcript length & Minimum of patient sentiment	0.328	1.389
Minimum of patient sentiment	0.225	1.253
Interval	0.078	1.081
Number of calls & interval	0.029	1.029
Bottom 5		
Total transcript length & Maximum of agent sentiment	- 0.073	0.929
Total transcript length & Interval	- 0.080	0.923
Total transcript length & Number of calls	- 0.102	0.903
Maximum of agent sentiment	- 0.161	0.851
Number of calls	- 0.430	0.650

As previously mentioned, combining metadata with topic information tends to yield better results than relying solely on topics. As such, we will be focusing on the topic + metadata model. Specifically, we are exploring the 5-topic + metadata model that demonstrated an AUC performance of 0.98. We intentionally selected this model due to its significant improvement in performance from 0.845 (as seen in the 4-topic + metadata model) to 0.98. The comprehensive OR scores for the models with different number of topics can be found in the Supplement (S3 Appendix)

Within the 5-topic + metadata model, we discovered that the OR ranges from 1.00 to 105.53 for variables positively associated with enrollment and from 0.20 to 0.98 for features negatively associated with enrollment. Table 4 displays the top and bottom five variables and their interactions with the highest and lowest OR values. It is interesting to note that Topic 5.5 (which is the fifth topic in the 5-topic + metadata model) has the highest odds ratio, while Topic 5.4 has the lowest. Factors that interact with Topic 5.5 have an odds ratio greater than 1, whereas the only factors that have an odds ratio greater than 1 when interacting with Topic 5.4 are Topic 5.5 and interval. The complete odds ratio value for the 5-topic + metadata model is available in the Supplemental document S4 Appendix Notably, the length of the transcript remains consistent among the top five, and the number of calls is consistent among the bottom five - just as we observed with the metadata-only model earlier.

**Table 4:** The top five variables exhibiting the highest coefficients and odds ratios, and the bottom five variables displaying the lowest coefficients and odds ratios within the 5-topic + metadata model.

Predictors	Coefficient	Odds Ratio (OR)
Top 5		
Topic 5.5^a^	4.659	105.528
Topic 5.1 & Topic 5.5	2.260	9.580
Total transcript length	1.619	5.049
Topic 5.5 & Topic 5.3	1.175	3.239
Topic 5.5 & Topic 5.4	1.051	2.861
Bottom 5		
Topic 5.2 & Topic 5.4	- 0.323	0.724
Number of calls	- 0.583	0.558
Topic 5.3	- 0.760	0.468
Topic 5.1 & Topic 5.4	- 1.184	0.306
Topic 5.4	- 1.630	0.196

^a^ Topic 5.5 indicates that this is topic 5 in the 5-topic + metadata model.

### Topic interpretation for 5-topic + metadata model

As an example of how to interpret the topics, we conducted an analysis of the 5-topic + metadata model, utilizing transcript examples with high topic proportions and FREX words. Note that the interpretation of the five topics in both the 5 topic-based model and the 5-topic + metadata model is the same because the same topics are used. Table 5 presents the corresponding topic number, interpretation, top 15 FREX words, interpretation description, and transcript content examples.

**Table 5:** Topic interpretation for the 5-topic model.

Topic number	Topic name	FREX words (top 15-words)	Description	Examples
5.1^a^	Greetings & insurance inquiry	awesome, helpful, wonder, manager, share, certainly, short, surprise, purpose, copay, particular, behalf, healthy, useful, whole	The initial patient greeting may vary in its content and tone during the conversation.	Agent (A): “Hello, this is calling on **behalf** of doctor [proper noun] ”.. “how are you doing today?.” Patient (P): “Pretty good..,” A:“ **Awesome**. Good to hear, doctor had wanted me to reach out just to share some information with you about a resource that she’s recommending”...
5.2	Patient’s overall condition	eat, kid, water, body, food, sick, die, leg, live, dog, blah, heart, grow, sit, light	Inquiry about the patient’s current health status and dietary habits. For instance, patients explain how their child primarily assists them in their daily life.	A: “..what’s your diet like take me through a day in your diet? ...” P : “... And so we try to **eat** a really good balanced dinner and, and uh so I mean a good no fried foods, no fatty, I can’t do a lot of that...”
5.3	Patient’s medication & diabetic condition	grid, sign, vitamin, check, symptom, everything, injection, since, watch, stable, last, difficulty, overall, pain	Inquiring about the patient’s medication regimen pertaining to their diabetic condition.	P: “For checking my, what, what is that?." A: “Your **grid**? So it’s a little **grid** with a dot in the middle that doctor wants you to look at each day to make sure your lines are still straight and there’s no curve lines..."
5.4	PCM service explanation	partner, program, extra, support, provide, basically, offer, office, interest, additional, enroll, guide, condition, train, qualify	Part of the agent’s script explains and offers the program to the patient.	A: ... “ Uh so this care coordinator is a certified professional who **partners** with doctor [PII] to **provide** you **extra support** over the phone.."
5.5	Setting up a call appointment	afternoon, week, morning, text, day, reminder, around, remind, prefer, o’clock, early, calendar, a.m., initial, cell	In this conversation, the agent offered to enroll the patient in the program. If the patient agrees, then the agent will attempt to schedule the patient for their first coaching call.	A: “I have **morning, afternoon** and I have early evening as well on those two days" P: “I can go into the next week if those two days don’t work..."

^a^ Topic 5.1 indicates that this is topic 1 in the 5-topics model.

As can be seen from Table 5, Topic 5.5 relates to setting up future appointments. Thus, we note that the high odds ratio for Topic 5.5. could suggest over fitting or be a potential signal for data contamination. Thus, we also run the 5-topic + metadata model *without* Topic 5.5. These results are presented in the Discussion section.

## Discussion

This study examined transcripts of calls made during the enrollment process to identify conversation topics and call metadata that may encourage patients to enroll in a telephone-based counseling program, PCM service. We found that the identified topics allow further understanding of the factors that lead to enrolling in the program. In addition, we observed that predictive performance could be increased by adding information extracted from the call transcript (unstructured data) to the call metadata (structured data).

Previous research suggests that combination of unstructured and structured data can improve the prediction performance. Ghassemi et al. [14], Feller et al. [15], and Dormosh et al. [16] found that incorporating unstructured data (i.e., text data) with structured data from electronic medical records (EMRs) such as age, sex, and medication can enhance predictive performance. Our results further support this finding. We found that incorporating unstructured data alongside structured data can increase a model’s predictive performance by providing additional information that may have been overlooked by structured data. However, different from the previous studies, our study utilized feature engineering to obtain our metadata as our structured data.

In this section, we will examine the insights that the metadata and the topic + metadata model provide, specifically for the 5-topic model. As mentioned earlier, we will not cover the topic-only model since the topic + metadata model performs better. We will first explore the call metadata factors that contribute to predicting enrollment. Afterward, we will discuss how topics can be used to support our concluding remarks.

### Call metadata

This section focuses on call metadata associated with patient enrollment in a PCM service, based on the odds ratio in Table 3. The metadata we analyzed includes transcript length, number of calls received, the time between calls (interval), maximum agent sentiment, and minimum patient sentiment. Based on our results, we found that patients are more likely to enroll in the program when they engage in longer conversations with agents, which results in a longer transcript length. However, it’s important to note that conversations with enrolled patients tend to be longer due to appointment setup discussions that aren’t relevant to patients who decline the program offer. Additionally, our findings suggest that the odds of enrollment decrease when transcript length interacts with other metadata, except when it interacts with the patient’s sentiment. In other words, the odds for a patient to enroll increase as the patient’s sentiment is increasing. This interaction indicates that it’s beneficial for agents to continue interacting with patients for a longer time as long as the patient’s sentiment is perceived to be favorable.

Furthermore, our result suggests that calling patients too often (i.e., increasing the “number of calls”) may not be an effective strategy for enrolling them in a program. Additionally, we found that longer time intervals between calls are associated with higher odds of enrollment. It is important to note that the number of calls and the interval between calls influence each other because taking longer breaks between calls translates to fewer calls made in the same timeframe.

Moreover, we found that the sentiments of patients and agents have a smaller impact on enrollment than other metadata. We observed that a higher sentiment expressed by patients was associated with enrollment. Conversely, the sentiment expressed by the agent was found to have a negative association with enrollment. Despite the odds ratio of patient and agent sentiment featuring in the top five and bottom five, respectively, its significance was comparatively lower than the factors of “total transcript length” and “number of calls”.

Interestingly, one key observation that persists from the metadata-based model in topic + metadata models is the consistency of the “total transcript length” and “number of calls” factors. The “total transcript length” consistently appears in the top five, while the “number of calls” appears in the bottom five across different predictive models with different numbers of topics. This consistent pattern highlights the significance of these variables.

### Topics

Our findings suggest that the identified topics from the NLP method, STM, can provide further insights about patients’ enrollment in addition to the structured data (i.e., call metadata). Topics notably enhance predictive performance in topic-based and topic+metadata models, compared to models relying solely on call metadata. To illustrate, we will discuss how the topics identified from the 5-topic model, along with call metadata, were associated with patient enrollment.

By analyzing conversation topics from the STM, we found that topics can provide extended insight into the factors that encourage patients to enroll. Specifically, we found that agents who offer to schedule the first coaching call appointment (Topic 5.5) have higher odds of patient enrollment than those who focus on other topics. While this may seem unsurprising, we were intrigued to discover that the topic centered around explaining the PCM service (Topic 5.4) had a negative association with patient enrollment unless agents also covered the topic of scheduling an appointment (Topic 5.5) and allowed for a longer interval between calls. This outcome was unexpected, given that agents are trained to provide comprehensive explanations and address any concerns to facilitate informed decision-making for patients.

**Fig 4:**
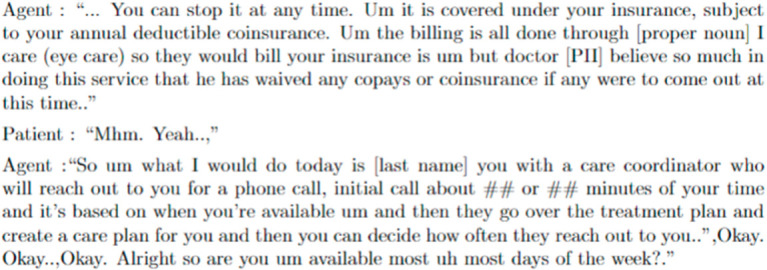
A transcript excerpt of an agent elaborating on a program and subsequently extending an offer to the patient.

To further understand the unexpected outcome, we conducted a detailed analysis of the topic of the program explanation (Topic 5.4). As previously mentioned, we found that agents who not only provide patients with information about the program (Topic 5.4) but also offer to schedule an appointment for the first telephone coaching (Topic 5.5) have higher odds of enrolling patients. This interaction indicates that offering to schedule an appointment after giving a brief explanation about the program might lead to patient enrollment, even if the patient has not yet decided to enroll in the program. The example of this case is shown in Fig 4. In this conversation, we observed that the agent intentionally offered to schedule an appointment after they explained the program. While it is possible that patients may enroll due to their interest after receiving a detailed explanation, program explanation by itself may not be enough to persuade patients to enroll, offering to schedule an appointment appears to also be effective in encouraging patients to enroll. Additionally, we found that agents who explain the program and have longer intervals between calls have higher odds of success than agents who call patients in shorter intervals. This implies that the agent should try to space out the calls, allowing the patient some time to contemplate the program and reconsider their decision. Patients may not appreciate receiving calls shortly after one another.

An alternative interpretation of Topic 5.5’s high influence is that it reflects post-enrollment discussion rather than a predictive factor for enrollment itself. If Topic 5.5 primarily identifies the scheduling of appointments after a patient has agreed to enroll, then our model would not be predicting enrollment based on pre-enrollment discourse but rather identifying a consequence of it. To investigate this, we re-ran the prediction model (5 Topics + Metadata Model) excluding Topic 5.5, henceforth referred to as the “Without Topic 5.5 Model." This ensured Topic 5.5’s complete exclusion from the Lasso regression. The model’s Area Under the Curve (AUC) only slightly decreased from 0.98 to 0.97. In other words, we still achieve strong predictive performance even when the proportion of the conversation related to setting up a call appointment is excluded from the analysis. The full set of coefficients and odds ratios associated with this model are provided in S6 Appendix.

Interestingly, the number of variables shrunk to zero by the Lasso model differed significantly between the two models. In the original “5 Topics + Metadata Model," 12 out of 56 variables were shrunk to zero, whereas in the “Without Topic 5.5 Model," only 2 out of 46 variables were shrunk. This considerable difference suggests that without Topic 5.5 exerting dominance over other variables as in the original model, many more factors influence enrollment. In particular, without Topic 5.5, Total Document Length emerged as the variable with the highest odds ratio, indicating that longer agent-customer interactions are associated with a higher likelihood of patient enrollment. Furthermore, in the absence of Topic 5.5, Topics 1 through 4 exhibited high odds ratios primarily when patients had been called multiple times (i.e., a high number of calls). Interestingly, these effective calls were not without substantial intervals; longer intervals between calls and higher customer sentiment were also associated with increased enrollment odds. The top 10 influential variables of the “Without Topic 5.5 Model" are presented in Table 6.

**Table 6:** The top ten variables exhibiting the highest coefficients and odds ratios within the 5-topic + metadata model *without* Topic 5.5 (i.e. Without Topic 5.5 Model).

Predictors	Coefficient	Odds Ratio (OR)
Total transcript length	1.899	6.681
Topic 5.3 & Number of calls	0.844	2.326
Topic 5.4 & Number of calls	0.606	1.833
Topic 5.1 & Number of calls	0.541	1.718
Topic 5.2 & Number of calls	0.536	1.709
(Intercept)	0.354	1.425
Minimum of patient sentiment	0.171	1.187
Total transcript length & Minimum of patient sentiment	0.108	1.114
Interval	0.079	1.082
Interval & Minimum of patient sentiment	0.058	1.060

Based on the above discussion, the PCM service can enhance call scripts and agent practices in multiple ways. Call scripts should include a section where agents actively propose scheduling a coaching call appointment at the end of each conversation. In terms of practice, agents should consider managing call duration, frequency, interval of time between calls, and patient sentiment. Agents may aim for slightly longer engagement during calls, identify an acceptable interval of time between contacts that patients tolerate well, and receive training on techniques to foster positive patient sentiment.

In summary, incorporating conversation topics can significantly improve the predictive accuracy of models when compared to those that solely rely on call metadata. Moreover, it was observed that extended engagement with patients often results in enrollment, while calling a patient too often can have a negative effect. Furthermore, discussing appointment scheduling during the call has a positive association with enrollment, whereas explaining the program during the call has a negative association. Interestingly, if the agent offers to schedule the first appointment with a care coordinator during the conversation, it may increase the odds of patient enrollment. Removing Topic 5.5 (appointment scheduling) from the model highlighted total document length and other topics in conjunction with number of calls and patient’s sentiment as key predictors.

## Limitation and future research

This study has some noteworthy limitations. First, we assumed the call transcripts contained reasons for enrollment in the program. However, external factors beyond the scope of the calls, such as personal circumstances, may exert influence on patients’ decisions regarding enrollment. The external factors could introduce complexity in determining why patients decline the offer during the call, and even when agents inquire for explanations, patients are not obligated to provide detailed responses. Moreover, patients may harbor concerns that the calls are phishing attempts, potentially explaining their hesitance to engage further. It’s noteworthy that the company has taken steps to address this by implementing caller ID, aiming to instill greater confidence in patients regarding the authenticity of the calls.

Second, the STM framework has limitations in capturing semantic nuances, synonyms, and antonyms due to its reliance on word occurrence. This poses a challenge for the framework in identifying phenomena such as the dual meanings of certain words like “may." However, this issue is not unique to STM but is prevalent in another topic model without word embeddings [32].

Third, while the transcript includes conversations about scheduling coaching calls which may signal enrollment, we still utilize these scheduling related exchanges to predict enrollment. However, recall that our enrollment outcome is based on whether the patient actually participated in a coaching call, and not just set up an appointment. Furthermore, we mitigate potential bias by converting these conversations into topic representations rather than using the raw text directly. We believe, this topic conversion process minimizes the impact of the scheduling-related content on the prediction by abstracting away the specific details of the conversation. While removing this part of the conversation could not be easily automated without potentially losing other important context or would need to be done manually, which is a time- consuming task, we re-evaluated the role of appointment scheduling by removing Topic 5.5 from the model. The “Without Topic 5.5 Model also achieved high AUC. This ensured that the performance of the model did not represent post-enrollment scheduling rather than a pre-enrollment influence.

Fourth, this research focused on conversations between PCM agents and DR patients specifically, which may be different than agent-patient interactions related to other diseases. This narrow focus, while allowing for in-depth analysis within this specific context, inherently limits the generalizability of our findings. The topics discussed in these interactions are likely highly tailored to the unique needs, concerns, and treatment protocols associated with DR. Consequently, the identified conversational topics, their relative importance, and their predictive power for patient enrollment may not be directly transferable to management of other diseases. However, we believe we have introduced a framework for analyzing real-world agent-patient consultations using mixed NLP and ML techniques and have demonstrated the potential of improving model performance by incorporating both text and other call metadata.

Future research could address these topic model limitations by exploring alternatives such as word embeddings, neural topic models, or models considering document structure, like Paragraph Vectors. These methods offer the potential for capturing richer semantic and contextual information in text data. In the context of call transcripts, while we were able to obtain good results with Topic 5.5 removed, developing auto-removal of the agent’s program explanation or scheduling the first appointment in call transcripts can improve topic extraction. Moreover, if we had access to linked records, incorporating additional variables about the patient (such as age, diabetic retinopathy (DR) severity, and HbA1c levels) or the agent (such as level of experience) into the model could further enhance our understanding of patient enrollment.

## Conclusion

The primary goal of this research is to investigate the factors that lead patients to enroll in a telephone-based counseling initiative (i.e., the PCM program). We analyzed the transcripts of the calls made during the enrollment process and transformed them into vector representations using an NLP method known as Structural Topic Modeling (STM). This method extracts a mixture of topics from each patient’s transcripts as a vector representation. We used these topics, along with call metadata, as transcripts’ features, to predict patient enrollment. We compared the predictive performance of three models, call metadata, topic-based, and combination of topic and metadata (i.e., topic+metadata) model.

Out of the three models, the topic + metadata model outperforms the other two models in distinguishing between patient enrollment and non-enrollment. By incorporating topic features from unstructured data to the call metadata as structured data, the predictive capability of the model was enhanced. Furthermore, adding topics to the prediction model can increase the model user’s comprehension of the relationship between unstructured data and factors associated with outcome prediction (i.e., patient enrollment).

Additionally, we consistently find across all models, that agents who explain the program and maintain longer intervals between calls have higher odds of patient enrollment. This finding indicates that waiting longer between calls gives patients time to consider the program. Furthermore, agents who engage in prolonged conversations tend to have higher odds of enrolling patients across different models. Conversely, calling patients too often indicates that this might not be the best approach.

The PCM service aims to offer patients extensive assistance in addressing their queries related to diabetic retinopathy. The findings in this study provide insights to agents in the enrollment process to evaluate their strategy in calling patients, which in turn can increase program participation among patients. The agent in the enrollment process is the first contact point between patients and the PCM service, which is a critical phase in determining whether the patient enrolls and eventually accesses the benefits of the program. The success of an agent to enroll a patient in the program is determined not only by external factors but also by the conversation that takes place during the call. This study attempts to capture this by analyzing the conversation during the call to draw insights. Therefore, this research has implications for improving the accessibility and effectiveness of the PCM service in promoting better outcomes for patients with DR.

## Supporting information

S1 File Agent’s guided script. This is the guided script for the agent to follow during the enrollment call.
(PDF)

S1 Appendix Topic proportion for 3 -15 topics model. (XLSX)

S2 Appendix 95% Confidence Intervals of 3-15 topics Topic+Metadata Model’s AUC. (XLSX)

S3 Appendix Odds ratio scores for topic + metadata models. This is the odds ratio score for the 3 -15 topics model in the topic+metadata model.
(XLSX)

S4 Appendix Odds ratio scores for topic + metadata models. This is the odds ratio score for the 5 topics model in the topic+metadata model.
(XLSX)

S5 Appendix Odds ratio scores of other metadata. (XLSX)

S6 Appendix Odds ratio scores Without Topic 5.5 model. This is the odds ratio score for the 5 topics model in the topic+metadata model with Topic 5.5 removed.
(CSV)
