# Supplementary material for: Predicting patient enrollment in a telephone-based principal care management service using topic modeling
Source: PLOS Digit Health. 2025 Sep 18;4(9):e0000992. doi: 10.1371/journal.pdig.0000992 (PMC12445525; doi:10.1371/journal.pdig.0000992)
Supplement: S1 File — This is the guided script for the agent to follow during the enrollment call. (PDF) [file pdig.0000992.s001.pdf]

Voicemail: Hi, this is (Your Name), from [redacted], a Care Coordination service that partners with (Dr. XYZ.) I am calling to share a resource Dr XYZ would like to offer you to help with your vision care in between appointments. Please call me back at (Phone Number) Again, my name is (Your Name) and you can reach me at (Phone Number). I look forward to hearing from you!

QUALIFICATION CHECK BEFORE CALL

Must have:

- Medicare or Medicare Replacement Plan
- A qualifying condition and level of severity that falls under the provider's preferences
- Active management provided by the participating physician
- Fulfilled a visit with their eye care provider in the previous year
- If Glaucoma diagnosis, patient must be on drops

Nice to have:

- Supplemental plan that will pick up out of pocket cost (Tricare does not)
- Multiple drops/medications to manage

SECTION I - INTRODUCTION

IMPORTANT: Follow the appropriate process for inbound and outbound Identity Verification

Hi, may I please speak with (Patient First and Last Name)?

[Patient responds]

Hi, (patient's name). This is (Your Name) from Lumata Health, a Care Coordination service that partners with (Dr. XYZ's) calling on a recorded line. How are you today?

[respond to patient]

I'm calling you because our partner Dr. XYZ wants to provide you with a care coordinator to provide a wide range of assistance, tailored to your individual eye care needs. Dr. XYZ is committed to your health and knows how chronic health issues can make getting to appointments, medications and other activities difficult.

SECTION II - ABOUT THE PROGRAM

Your care coordinator is a certified professional who partners with Dr. XYZ to provide you extra support **over the phone** in between your appointments to help guide you towards better eye health. We are here to address questions and concerns about your treatment, provide education on (patient condition), help you monitor your vision for changes, and make sure you have the right support in place to preserve your vision.

We can be flexible on how often we talk. Our phone calls can be as frequent or infrequent as you'd like, and you can stop at any time. Care Coordination is covered by Medicare and is subject to your annual deductible and coinsurance (usually 20%), but if you have a secondary or supplemental insurance plan, your coinsurance may be covered.

- If a patient asks about insurance coverage specifics: A claim will be submitted every time we spend at least 30 minutes managing your care within a calendar month. So let's say we talk every 2 months, then you'd have around 6 claims submitted in a year for these encounters. Since you (do/don't) have secondary coverage, this program should (be no cost to you/include your typical cost sharing at 20%, which usually amounts to less than $10-12).

- The practice will bill your insurance every time we spend at least 30 minutes providing care coordination in a calendar month.Care coordination services like this actually save medicare an average of $70 per patient per month by keeping your disease from getting worse which would require more expensive treatment.

SECTION IIA- ENROLLMENT

(Enrollment Specialist): **So let's schedule time for you to work with your Care Coordinator.** What days/times are you most available for the Care Coordinator to call you? (document response in "General Availability" field) .

- If patient doesn't agree → Okay, no problem. What concerns do you have about working with a Care Coordinator so I can update Dr. XYZ?
- If agrees → **document response and** prepare them for the call with Coordinator

Great, I have you scheduled for around ___ AM/PM on ____ (date).Your care coordinator will call you to ask a few questions related to your (Patient Condition) care and create a care plan with information personalized to you and your needs. The care plan is meant to be a helpful resource for you. It takes about 10 minutes or so to complete. We will reach out to remind you of the scheduled call. Would you like the reminder to be a text or phone call?
